# Latina Health in the United States: A Public Health Reader

**Published:** 2004-12-15

**Authors:** Gloria L.A. Beckles

**Affiliations:** Division of Diabetes Translation, National Center for Chronic Disease Prevention and Health Promotion, Centers for Disease Control and Prevention Atlanta, GA

*Latina Health in the United States: A Public Health Reader* is a collection of articles drawn from journals and books published in the last decade about the health status and health needs of Latin American adolescent and adult females in the United States. In the preface, the editors state that the goals of the anthology are to identify a number of critical issues of importance and relevance to Latina health; to present an overview of the existing literature on Latina health; to highlight the leading indicators of morbidity and mortality affecting various subgroups of Latinas; and to identify gaps in research, policy issues, program planning, and practice. To achieve these goals, the editors organized the book's 29 chapters into nine parts that explore the population's demographics; risk factors, socioeconomic disparities, and race/ethnic disparities among Latinas; sexual and reproductive health issues; chronic conditions such as heart disease, cancer, and diabetes; alcohol, tobacco, other drug use, and mental health; patterns of risk behaviors among Latina adolescents; and the health needs of rural and migrant workers.

In Part 1, the editors describe the process used to identify the essential topics for inclusion in the book. Content experts were interviewed, morbidity and mortality data were reviewed, and a comprehensive review of papers published during 1984–2002 was undertaken to identify the extent to which published research reflects the health needs of Latinas. The editors conclude that research on Latinas has several limitations, including lack of generalizability, small sample size, misclassification bias (when Spanish surnames are used to identify sampling frames), and lack of comparison groups. Furthermore, they report that few journals publish studies related to Latina health issues. In Parts 2 through 6, contributors document the principal health conditions that beset Latinas and the persistent racial and ethnic differences in biological factors, behavioral risk factors, and use of health services. Parts 3, 7, and 9 focus on the health and health care experience of women at different life stages (adolescence, the reproductive years, and midlife).

*Latina Health in the United States* is primarily intended for four groups of people: students, practitioners, decision makers, and researchers who seek to address the challenge of the growing health disparities among communities of color. The book should be required reading in all schools of public health; it synthesizes and presents in a single source the major issues on the health of women of Latin American origin. For public health practitioners and decision makers, it should be a necessary general reference because it calls attention to issues that have important implications for health policy and planning of programs and services (e.g., the heterogeneity in geographic origins of Latinas, the lack of data to provide timely assessment and monitoring of the health status of Latinas). Additionally, the population dynamics (e.g., high fertility and immigration rates, regional concentration) that lead to rapid population growth raise the question about the ability of the health care system, at least regionally, to meet the current and future health needs of Latina women.

The gaps in knowledge of Latina health issues documented by the articles in the book pose a challenge to researchers. In particular, the articles demonstrate the necessity to understand the mechanisms and pathways by which structural and contextual factors (e.g., poverty, low-wage employment, social capital, cultural norms) may protect against or determine the health deficits that often result from international migration and acculturation. The findings from such research could be used to develop intervention strategies to preserve protective behaviors and to contribute to risk reduction in Latinas and other women.

Overall, the editors have realized several of their stated goals. *Latina Health in the United States* is a much needed and timely collection that chronicles a wealth of information — information that can sharpen the focus and guide the direction of efforts to improve the health of Latinas.

